# *Lactobacillus rhamnosus* GG-supplemented formula expands butyrate-producing bacterial strains in food allergic infants

**DOI:** 10.1038/ismej.2015.151

**Published:** 2015-09-22

**Authors:** Roberto Berni Canani, Naseer Sangwan, Andrew T Stefka, Rita Nocerino, Lorella Paparo, Rosita Aitoro, Antonio Calignano, Aly A Khan, Jack A Gilbert, Cathryn R Nagler

**Affiliations:** 1 Department of Translational Medical Science, Section of Pediatrics, European Laboratory for the Investigation of Food-Induced Diseases, University of Naples, Federico II, Naples, Italy; 2 Institute for Genomics and Systems Biology, Department of Biosciences, Argonne National Laboratory, Argonne, IL, USA; 3 Committee on Immunology and Department of Pathology, University of Chicago, Chicago, IL, USA; 4 Department of Pharmacy, University of Naples Federico II, Naples, Italy; 5 Toyota Technological Institute at Chicago, Chicago, IL, USA; 6 Department of Surgery, University of Chicago, Chicago, IL, USA; 7 Department of Ecology and Evolution, University of Chicago, Chicago, IL, USA

## Abstract

Dietary intervention with extensively hydrolyzed casein formula supplemented with *Lactobacillus rhamnosus* GG (EHCF+LGG) accelerates tolerance acquisition in infants with cow's milk allergy (CMA). We examined whether this effect is attributable, at least in part, to an influence on the gut microbiota. Fecal samples from healthy controls (*n*=20) and from CMA infants (*n*=19) before and after treatment with EHCF with (*n*=12) and without (*n*=7) supplementation with LGG were compared by 16S rRNA-based operational taxonomic unit clustering and oligotyping. Differential feature selection and generalized linear model fitting revealed that the CMA infants have a diverse gut microbial community structure dominated by Lachnospiraceae (20.5±9.7%) and Ruminococcaceae (16.2±9.1%). *Blautia, Roseburia* and *Coprococcus* were significantly enriched following treatment with EHCF and LGG, but only one genus, *Oscillospira*, was significantly different between infants that became tolerant and those that remained allergic. However, most tolerant infants showed a significant increase in fecal butyrate levels, and those taxa that were significantly enriched in these samples, *Blautia* and *Roseburia*, exhibited specific strain-level demarcations between tolerant and allergic infants. Our data suggest that EHCF+LGG promotes tolerance in infants with CMA, in part, by influencing the strain-level bacterial community structure of the infant gut.

## Introduction

The prevalence of allergic responses to food has been experiencing an unprecedented increase in developed societies, rising by as much as 20% in a recent 10-year period (Branum and Lukacs, 2009; Osborne *et al.*, 2011; Wang and Sampson, 2011; Prescott *et al.*, 2013). Genetic variation alone cannot account for a dramatic increase in disease prevalence over such a short time frame. Emerging evidence suggests that twenty-first century environmental interventions, including widespread antibiotic use, consumption of a high-fat/low fiber diet, elimination of previously common enteropathogens (including *Helicobacter pylori* and helminthic parasites), reduced exposure to infectious disease, Caesarean birth, and formula feeding, may have perturbed the mutually beneficial interactions established over millions of years of co-evolution with the bacteria that comprise our commensal microbiota (Cho and Blaser, 2012). This dysbiosis can predispose genetically susceptible individuals to allergic disease (reviewed in ref. Feehley *et al.*, 2012). Cow's milk allergy (CMA) is one of the most common food allergies of infancy and early childhood with an estimated prevalence of 2–3% worldwide (Sicherer, 2011). We have demonstrated that dietary management with a formula containing an extensively hydrolyzed form of the cow's milk protein casein (EHCF), supplemented with the probiotic *Lactobacillus rhamnosus* GG (LGG), results in a higher rate of tolerance acquisition in infants with CMA than in those treated with EHCF without supplementation or with other non-casein-based formulas (Berni Canani *et al.*, 2012, 2013). However, the mechanistic basis for this effect is not known. We hypothesized that it is attributable, in part, to an influence of this dietary intervention on the composition of the gut microbiota. To test this hypothesis, we performed 16S ribosomal RNA (rRNA)-based amplicon sequencing and oligotyping analysis on stool samples collected from healthy infants and from CMA infants before and after treatment with EHCF with or without supplementation with LGG.

## Materials and methods

### Patient enrollment and sample collection

Infants invited to participate in the study were referred to a tertiary pediatric allergy center (Pediatric Food Allergy Unit at the Department of Translational Medical Science of the University of Naples ‘Federico II') for a full diagnostic work-up for suspected CMA. All patients were still receiving cow's milk protein (mainly from formula feeding) at the time of enrollment and first stool sampling. The inclusion criteria were infants aged 1–12 months with a recent strong suspicion of IgE-mediated CMA but still receiving cow's milk protein. Diagnosis of IgE-mediated CMA was based on clinical history, the results of a double blind placebo-controlled oral food challenge, and the level of serum-specific anti-cow's milk protein IgE (Berni Canani *et al.*, 2011). Patients administered pre- or probiotic products and/or antibiotics in the previous 4 weeks, and patients with a history of cow's milk-induced anaphylaxis, eosinophilic disorders of the gastrointestinal tract, food protein-induced enterocolitic syndrome, concomitant chronic systemic diseases, congenital cardiac defects, active tuberculosis, autoimmune diseases, immunodeficiency, chronic inflammatory bowel diseases, celiac disease, cystic fibrosis, metabolic diseases, lactose intolerance, malignancy, chronic pulmonary diseases or malformations of the gastrointestinal tract were excluded. Fecal samples were collected at baseline before diet therapy from patients with a confirmed diagnosis of IgE-mediated CMA according to standardized criteria (Berni Canani *et al.*, 2011). Following the initial visit, patients were treated by dietary management with a commercially available extensively hydrolyzed casein formula (EHCF, Nutramigen, Mead Johnson, Rome, Italy) either with or without supplementation with LGG (at 4.5 × 10^7^–8.5 × 10^7^ colony-forming units per gram of powder (Berni Canani *et al*, 2012). A second fecal sample was obtained after 6 months. Samples obtained from healthy (non-allergic) infants who visited the study clinic as part of a vaccination program served as controls. These subjects were not at risk for atopic disorders and their clinical history was negative for any allergic condition. The study was conducted in accordance with the Declaration of Helsinki and approved by the Ethics Committee of the University of Naples ‘Federico II'.

### Fecal DNA isolation and 16S rDNA sequencing

Feces were collected and frozen at −20 °C immediately after excretion. To isolate DNA, 100–300 mg of fecal material was bead beaten before extraction with the QIAamp DNA stool mini kit (Qiagen, Hilden, Germany). 16S V4-region amplicon libraries were produced using previously described primers and sequenced using the Illumina MiSeq platform (150 bp × 2) at Argonne National Laboratory's Biosciences Sequencing Core facility (Caporaso *et al.*, 2011). Bacterial load was determined by quantitative PCR using a standard curve derived from a plasmid containing a single copy of the 16S rRNA encoding gene (Stefka *et al.*, 2014). Sequence data have been deposited in MG-RAST (http://metagenomics.anl.gov) under accession numbers 4571868.3–4571924.3 and project number 10023.

### Bioinformatics analysis

Paired end reads were quality trimmed and processed for operational taxonomic unit (OTU) clustering using UPARSE pipeline (Edgar, 2013), set at 0.97% identity cutoff. Taxonomic status was assigned to the high-quality (<1% incorrect bases) candidate OTUs using the ‘parallel_assign_taxonomy_rdp.py' script of QIIME software (Caporaso *et al.,* 2010). Multiple sequence alignment and phylogenetic reconstruction were performed using PyNast and FastTree (Caporaso *et al.*, 2010). Phyloseq package (McMurdie and Holmes, 2013) was used for the detailed downstream analysis on a rarefied abundance matrix. This matrix was processed to remove OTUs containing less than five reads to reduce the PCR and sequencing based bias; then, the OTU table was rarified to the minimum numbers of reads present in the smallest library (3746 reads). We used the oligotyping pipeline (Eren *et al.*, 2013) to identify the sub-OTU level differences in the top five most differentially abundant genera, that is, *Roseburia, Blautia, Coprococcus, Faecalibacterium* and *Bifidobacterium*, as predicted by MetagenomeSeq (Paulson *et al.*, 2013).

### Statistical analysis

We used MetagenomeSeq (Paulson *et al.*, 2013) software to determine the differentially abundant OTUs, families, and genera, present across all groups. We also used nonparametric Kruskal–Wallis H-test (*post hoc* Tukey Kramer tests, Bonferroni multiple test correction) for multi-group comparisons. Two-group and two-sample comparisons were performed using Welch's *t*-test and Fisher's exact *t*-test, respectively (two-sided with Bonferroni correction). We compiled a de-identified metadata table containing all of the clinical and demographic data for each infant in this study (Supplementary Table 1). We then used a generalized linear regression model (GLM) to examine the contribution of seven measurable features from the patient demographic data ('mode of birth', 'age at introduction of solid foods', 'age at initial sampling', 'sex', 'body weight', 'duration of exclusive breastfeeding' and ‘health status' (that is, healthy or CMA)) on the bacterial abundance of the differentially abundant bacterial families and strains as predicted by oligotyping. A GLM model was constructed and validated using rms and ResourceSelection (Lele, 2009) packages, respectively. We modeled bacterial abundances using a binomial distribution with a logit link function. To examine whether fecal butyrate levels correlated with bacterial diversity (Shannon diversity index) and evenness (Pielou's evenness index) and oligotype abundance patterns across multiple groups (that is, healthy, CMA, EHCF and EHCF-LGG), we calculated the Spearman correlation using the cor.test function implemented in R (http://www.r-project.org/).

### Determination of fecal butyrate concentration

Frozen feces weighing 1 g were diluted with saline, vortexed and centrifuged at 13 000 r.p.m. for 10 min in 2-ml tubes. The supernatants were filtered (0.45 μm) and used as the fecal extracts, which were stored at −20 °C until analysis. To determine fecal butyrate concentration, frozen fecal extracts were acidified with 20 μl 85% phosphoric acid and 0.5 ml ethyl acetate, mixed, centrifuged at 14 000 r.p.m. for 1 h and extracted in duplicate. A quantity of the pooled extract containing the acidified butyrate was transferred into a 2-ml glass vial and loaded onto an Agilent Technologies (Santa Clara, CA, USA) 7890 gas chromatograph (GC) system with automatic loader/injector. The GC column was an Agilent J&W DB-FFAP (Agilent Technologies) with the length 30 m, internal diameter 0.25 mm and film thickness 0.25 μm. The GC was programmed to achieve the following run parameters: initial temperature 90 °C, hold 0.5 min, ramp 20 °C min^−1^, final temperature 190 °C, total run time 8.0 min, gas flow 7.7 ml min^−1^ split less to maintain 3.26 p.s.i. column head pressure, septum purge 2.0 ml min^−1^. Detection was achieved using a flame ionization detector. Peaks were identified using a mixed external standard and quantified by peak height/internal standard ratio.

## Results

### The gut microbiota of cow's milk allergic infants exhibits significantly increased diversity and altered composition

**Figure 1:**
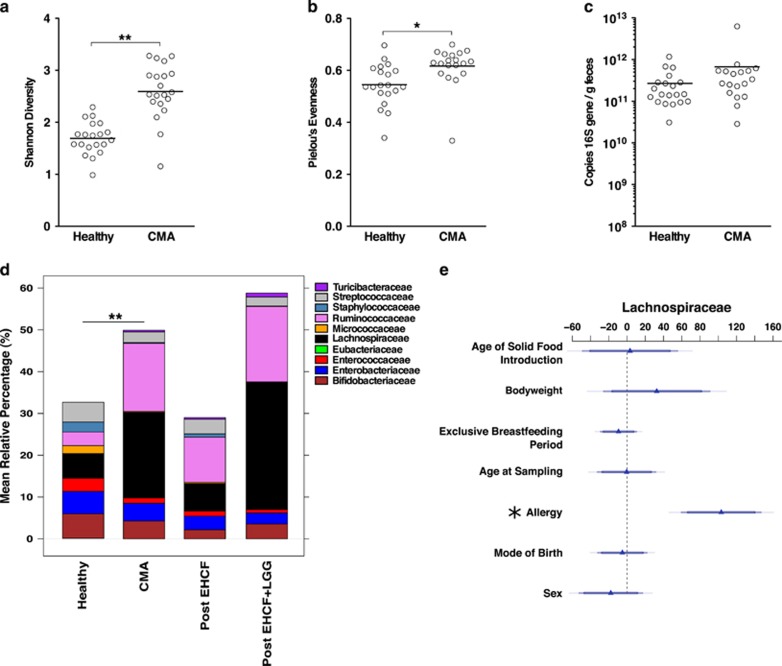
Significantly diverse bacterial community dynamics across cow's milk allergy and its treatment. (**a**) Shannon diversity, (**b**) Pielou's evenness and (**c**) bacterial load in fecal samples from each healthy (*n*=20) or age-matched pre-treatment cow's milk allergic (CMA, *n*=18–19) patients at diagnosis. (**d**) Family level differential abundance across healthy, CMA pre-treatment and treated groups, as computed by MetagenomeSeq. Families depicted are those determined to be differentially abundant. (**e**) Generalized linear model fitting of patient demographic information across relative abundance of family Lachnospiraceae. Parallel *x* axis represents the relative contribution value of every factor, as predicted by the GLM model. *P<0.05, **P<0.0001, by two-sided *t*-test or by GLM model.

A fecal sample was obtained before diet therapy from 19 patients with IgE-mediated CMA. During the same study period, fecal samples were also obtained from 20 age, sex and body weight-matched healthy infants enrolled in a vaccination program. All study subjects were breastfed for <1 month after birth and were still receiving a formula containing cow's milk proteins at the time of enrollment and first fecal sampling. A second fecal sample was obtained from each of the CMA infants after 6 months of treatment with EHCF with or without supplementation with LGG. The demographic and clinical characteristics of the study population are summarized in Table 1. A metadata table containing all of the demographic and clinical information for each patient in this study is provided in Supplementary Table 1. To compare the fecal microbiota of healthy and CMA infants, we generated 1.7 million 16S rRNA V4 amplicon sequences, which following quality control, clustered into 592 OTUs (97% nucleotide identity). Ordination and classification independent analyses of overall bacterial community structure demonstrated that allergic infants were significantly more diverse than age-matched healthy controls (Shannon's index, healthy=1.7±0.8 vs CMA=2.6±0.4; Figure 1a), and also significantly more even (Pielou's evenness; healthy=0.52±0.2 vs CMA=0.6±0.3; Figure 1b). Bacterial 16S rRNA abundance was similar in all samples (Figure 1c).

Taxonomic assignment revealed marked differences between healthy and allergic infants. CMA infants had a significant reduction in Bifidobacteriaceae, Streptococcaceae, Enterobacteriaceae and Enterococcaceae, and were significantly enriched for Ruminococcaceae (16%) and Lachnospiraceae (20.5% Figure 1d). The CMA infant gut microbiota comprised 73% Bacteroidetes and Firmicutes taxa, which are also known to dominate in the adult gut (The Human Microbiome Project Consortium, 2012). Genus-level analysis revealed a significant enrichment in CMA infant samples of *Ruminococcus* and *Faecalibacterium*, and a significant reduction in *Bifidobacterium* and *Escherichia* (Welch's *t*-test) compared with healthy samples (Supplementary Table 2).

To examine whether these significant differences could be explained by demographic variables, we applied a GLM for seven features (Supplementary Table 1) fitted against the relative abundance of the significantly different taxa. Health status (that is, healthy or CMA) was the single largest significant contributor to the differential abundance of Lachnospiraceae (*P*=5.74e−05; Figure 1e), Ruminococcaceae (*P*=0.00144; Supplementary Figure S1A), Enterobacteriaceae (*P*=0.0003; Supplementary Figure S1B) and Streptococcaceae (*P*=0.00198; Supplementary Figure S1C). It was also the second largest contributor to Bifidobacteriaceae (*P*=0.0034, Supplementary Figure S1D). Mode of birth and gender did not significantly contribute to these differences. However, body weight and age at initial sampling were significantly associated with the abundance of Enterobacteriaceae (*P*<0.0034, Supplementary Figure S1B) and Bifidobacteriaceae (*P*<0.00633, Supplementary Figure S1D), respectively. We validated the GLM model by fitting these features against genus-level abundances for *Faecalibacterium*, (*P*<0.0001, Supplementary Figure S2A), *Ruminococcus* (*P*<0.0029, Supplementary Figure S2B) *Escherichia* (*P*<0.0071, Supplementary Figure S2C) and *Bifidobacterium (P<0.0031,* Supplementary Figure S2D). These observations suggested that CMA, more than any other measured demographic variable, was the most important factor affecting the significantly different components of the gut microbiome.

### Treatment with LGG-supplemented EHCF increases the relative abundance of butyrate-producing bacteria and fecal butyrate levels

**Figure 2:**
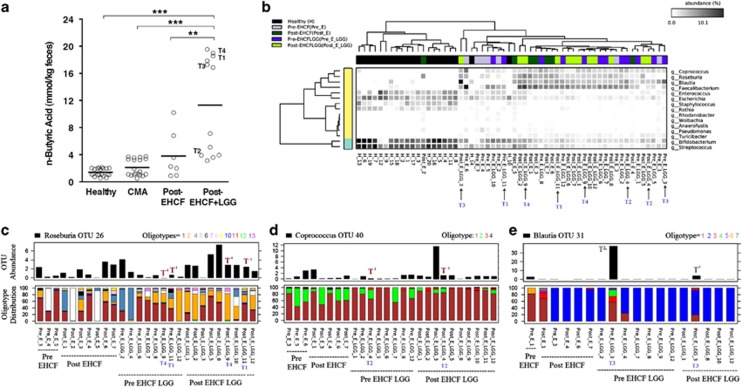
Microbial community dynamics of fecal samples from cow's milk allergic infants (CMA) before and after treatment. (**a**) Butyrate (n-butyric acid) concentration in fecal samples from healthy patients (*n*=20), or from CMA patients before (CMA, *n*=19) and after treatment (post-EHCF, *n*=7; post-EHCF+LGG, *n*=12). (**b**) Differential features (genera) selection analysis across healthy, CMA pre-treatment and treated groups (EHCF and EHCF+LGG). Abundance matrix was processed using Kruskal–Wallis H-test (*post hoc* tests=Tukey Kramer, multiple test correction=Bonferroni) with hierarchical clustering of both rows (genera; *y* axis; clusters are represented by color bars) and columns (samples; *x* axis; clustering is performed with ‘average' linkage, using Bray–Curtis' distance for genera and ‘correlation' for samples). The heatmap key shows percent relative abundance. Oligotyping analysis reveals strain-level differential selection in (**c**) *Roseburia*, (**d**) *Coprococcus* and (**d**) *Blautia* enriched across CMA and EHCF+LGG samples. Samples from EHCF+LGG-treated infants determined to be tolerant after double blind placebo-controlled oral food challenge analysis are labeled as ‘T'. **P< 0.05, ***P < 0.001, by Kruskal–Wallis H-test.

Acquisition of tolerance was evaluated by double blind placebo-controlled oral food challenge following 12 months of treatment. In total, 5 out of 12 (42%) EHCF+LGG-treated infants developed tolerance to cow's milk proteins, whereas all (7/7) of the EHCF-treated infants remained allergic (*P*=0.1, Fisher's exact test). We hypothesized that EHCF+LGG promotes tolerance to cow's milk proteins in part by altering gut microbial community structure. The fecal concentration of butyrate was significantly greater in CMA infants treated with LGG-supplemented EHCF, when compared with those treated with EHCF alone (Figure 2a). When we examined the samples from pre- and post-EHCF-LGG treatment, *Blautia*, *Roseburia* and *Coprococcus* were significantly enriched post-treatment (*P*<0.01; Supplementary Table 2). However, *Roseburia* was also significantly enriched post-treatment with EHCF alone (*P*<0.01). There were also genus-level differences post-treatment between the EHCF and EHCF-LGG groups; two-group analysis revealed *Roseburia* and *Anaerofustis* as significantly enriched in the Post-EHCF-LGG group (Supplementary Table 2).

The infants treated with EHCF+LGG had a bi-modal distribution of butyrate production post-treatment that was not seen in the children treated with EHCF alone (Figure 2a). *Bacteroides* was significantly reduced in abundance in the high butyrate group, whereas known butyrate producers, *Faecalibacterium*, *Blautia*, *Ruminococcus* and *Roseburia* were significantly enriched in high butyrate samples (Supplementary Table 2). In addition, the alpha diversity and evenness of the microbial community had a significantly positive correlation with the quantity of butyrate post-treatment (Supplementary Figures S3A and B).

Post-EHCF-LGG samples (*n*=12) were divided into two groups; tolerant (*n*=5) and allergic (*n*=7). Phylogenetically independent beta diversity analysis (OTUs) clearly highlighted the inter- and intragroup divergence between samples from tolerant and allergic infants (Supplementary Figure S3C). Interestingly, the tolerant group had a significantly (*P*<0.0032, Welch's *t*-test) greater average butyrate concentration (12.52±0.32 mmol kg^−1^) than those infants who remained allergic (10.32±0.3 mmol kg^−1^). In addition, 80% (4/5) of the tolerant samples had higher butyrate concentrations with respect to their paired samples in Pre-EHCF-LGG group (Supplementary Figure S3B). However, although tolerant sample 5 (T5) did show a significant increase in butyrate following treatment, the quantity of butyrate was still low (Supplementary Figure S3B). Interestingly, we observed that tolerant samples with high levels (>10 mmol kg^−1^) of fecal butyrate (T1, 3 and 4) had a greater relative abundance of *Roseburia* and *Blautia* compared with samples with low levels (<6 mmol kg^−1^) of fecal butyrate (T2 and 5; Supplementary Table 2). *Bacteroides* was enriched in the low level (<6 mmol kg^−1^) butyrate samples. Two-group analysis (two-sided Fisher's exact *t*-test with Storey's false discovery rate correction) was performed between samples from tolerant and allergic infants before and after EHCF-LGG treatment. There were no significantly different genera before EHCF+LGG treatment that identified infants who eventually became tolerant to cow's milk. In fact, the only genus that was significantly different between tolerant and allergic infants post-treatment with EHCF+LGG was *Oscillospira*, which was enriched in allergic samples (Supplementary Table 2). Owing to its low abundance (<0.1%) we were not able to perform subsequent oligotyping analysis (see below) on *Oscillospira.*

### Strain-level differences between tolerant and allergic infants after treatment may have a role in tolerance acquisition

Although only *Oscillospira* was significantly different between tolerant and allergic infants, the fact that *Blautia, Roseburia* and *Coprococcus* were significantly different pre- and post-treatment with EHCF+LGG (Figure 2b), and *Blautia* and *Roseburia* were significantly enriched in samples from tolerant infants with higher concentrations of fecal butyrate, led us to hypothesize that tolerance may be associated with the acquisition of specific strains of these genera in tolerant individuals. We explored whether there was any correlation between the abundance of strains of these genera (100% nucleotide clustered oligotypes), the increase in fecal butyrate concentration, and the acquisition of tolerance by the infants. Oligotyping of *Roseburia*, *Coprococcus* and *Blautia* (Figures 2c–e, respectively) revealed demarcations among the treatment groups, especially across the sample pairs representing CMA tolerant patients. Significant differences in oligotype abundances between samples were analyzed using a two-sample test (Fisher's exact *t*-test with two-sided with Bonferroni multiple test correction).

The four tolerant patients for whom oligotypes of these genera could be detected are marked ‘T' in Figure 2 (T1–4). *Roseburia* OTU 26 disassociated into 13 oligotypes that each presented different patterns (Figure 2c), similar to *Coprococcus* OTU 40, which disassociated into four oligotypes (Figure 2d). However, *Blautia* OTU 31, which disassociated into seven oligotypes, was generally at very low relative abundance, except in samples from T3, where it had significantly lower relative abundance post-treatment (Figure 2e).

The strain-level differential abundance patterns of *Roseburia* and *Coprococcus* between the tolerant and allergic groups, and between high and low butyrate-producing subgroups of post-EHCF+LGG samples were assessed. In addition, we also analyzed how strain-level patterns varied across tolerant samples before and after EHCF+LGG treatment. Strikingly, the total strain profile of *Roseburia* (*R*^2^=0.90) and *Coprococcus* (*R*^2^=0.94) was very similar in both the tolerant and allergic groups (*P*<0.001, Welch's *t*-test). However, *Roseburia* oligotype 2 and *Coprococcus* oligotype 1 were significantly enriched in the tolerant group (Supplementary Table 2). The high and low butyrate-producing groups revealed lower levels of community (strain level) overlap, when compared with the tolerant- allergic analysis (*Roseburia* *R*^2^=0.64 and *Coprococcus* *R*^2^=0.63). The relative abundance of *Roseburia* oligotype 2 and *Coprococcus* oligotype 1 were still significantly enriched in the high butyrate-producing group (Supplementary Table 2). Interestingly, two-group analysis of tolerant samples before and after treatment with EHCF+LGG revealed a significant enrichment in *Roseburia* oligotype 2 and *Coprococcus* oligotype 1 post-treatment (Supplementary Table 2).

Fecal butyrate concentrations were positively correlated with the abundance of *Roseburia* oligotype 2 (*R*^2^=0.5, *P*<0.00061) and *Coprococcus* oligotype 1 (*R*^2^=0.36, *P*<0.18). Our data suggest that LGG treatment enhances acquisition of tolerance to cow's milk, in part, by changing the strain-level community structure of taxa with the potential to produce butyrate. However, this strain-level correlation analysis would be best validated with wider sampling size including longitudinal time series events.

## Discussion

The microbiota of CMA infants was significantly more diverse than that of healthy controls. Bacterial families characteristic of the healthy infant gut (notably, Enterobactericeae and Bifidobacteriaceae) were significantly less abundant in the CMA gut, and were replaced by an increase in Lachnospiraceae and Ruminococcaceae, representing an emergence of Firmicutes (particularly, Clostridiales). *Blautia, Roseburia* and *Coprococcus* were significantly enriched following treatment with EHCF and LGG, but only one genus, *Oscillospira*, was significantly different between infants that became tolerant and those that remained allergic. However, most tolerant infants showed a significant increase in fecal butyrate levels, and those taxa that were significantly enriched in these samples, *Blautia* and *Roseburia*, exhibited specific strain-level demarcations between tolerant and allergic infants.

Whether or not differences in the composition of the microbiota (particularly abundance of Bifidobacteriaceae) precede the development of atopy, as suggested by other reports (Bjorksten *et al.*, 2001; Kalliomaki *et al.*, 2001a; Penders *et al.*, 2013) is not addressed in the current study, as the first fecal sample was collected after the onset of CMA signs and symptoms. However, as we have recently reviewed, increasing evidence supports a role for the microbiota in sensitization to food allergens, where the use of antibiotics, anti-bacterial agents and disruptions in fecal-associated community structure correlate with an elevated risk of disease (Berni Canani *et al.*, 2015).

In the current report, the study cohort was selected based upon a direct examination of fecal samples obtained from CMA infants at diagnosis. The use of a local Italian population with limited racial and ethnic diversity and similar environmental influences (for example, diet) is likely to have minimized interindividual variation in our study population. Using an unbiased nonparametric statistical approach, we demonstrated that allergic status was the most significant correlative factor for the composition of the gut microbiota in CMA infants. Several studies have suggested that providing combined antenatal and postnatal supplementation with the probiotic LGG to infants at risk for atopic diseases protects against subsequent allergic sensitization (Kalliomaki *et al.*, 2001b; Kalliomaki *et al.*, 2003; Huurre *et al.*, 2008). LGG may contribute to acquisition of tolerance to food allergens through the modulation of cytokines that influence gut permeability, thereby limiting the immune system's exposure to dietary allergens (Pohjavuori *et al.*, 2004; Ghadimi *et al.*, 2008; Mileti *et al.*, 2009; Donato *et al.*, 2010). Treatment with a LGG-supplemented formula has previously been associated with alterations in the composition of the gut microbiota (Cox *et al.*, 2010). Although in the present study it is not possible to determine the mechanism by which LGG treatment influences microbial community composition and structure in these samples, other work has begun to suggest potential ways by which probiotics structure the host–gut ecosystem to affect microbial ecology. For example, introduction of *Bacteroides fragilis* into the gut environment of a mouse model of autism influenced the microbial community structure by producing a biofilm that was potentially associated with the intestinal wall (Hsiao *et al.*, 2013). This interaction is likely to have shaped the microbial community by altering the host immune response, changing the metabolic interaction space in the gut, and altering the physical environment.

Although further analysis will be required to elucidate the mechanisms for their selection the observed increase in the relative abundance of specific strains of *Roseburia* and *Coprococcus* in CMA infants successfully treated with EHCF-LGG is nonetheless intriguing. These genera belong to the Clostridiales, which comprise a large, heterogeneous bacterial order (Nava and Stappenbeck, 2011; Nagano *et al.*, 2012). Short chain fatty acid (SCFA) production, particularly that of butyrate, is enriched within Clostridium cluster XIVa, (Sokol *et al.*, 2008; Louis and Flint, 2009; Sokol *et al.*, 2009; Miquel *et al.*, 2013; Van den Abbeele *et al.*, 2013). Butyrate is the preferred energy source for colonocytes and is often considered a sensor of intestinal health (Miquel *et al.*, 2013). Both bacterial abundance and SCFA production are sensitive to dietary manipulation (Duncan *et al.*, 2007; Louis and Flint, 2009; De Filippo *et al.*, 2010). Indigenous clostridial strains from clusters IV, XIVa and XVIII isolated from both mice (Atarashi *et al.*, 2011) and humans (Atarashi *et al.*, 2013) are among the most potent inducers of Foxp3^+^ regulatory T cells in the colonic lamina propria. Bacteria-produced SCFAs critically regulate both the proportions and functional capabilities of colonic regulatory T cells (Arpaia *et al.*, 2013; Smith *et al.*, 2013) and this phenomenon has been specifically linked to butyrate production by spore-forming Clostridiales (Furusawa *et al.*, 2013). We have recently described a novel mechanism by which Clostridia regulate innate lymphoid cell function to alter epithelial permeability and reduce allergen uptake into the systemic circulation (Stefka *et al.*, 2014). Preliminary data from our laboratory links butyrate, but not other SCFAs, to regulation of epithelial barrier function (Feehley *et al.,* personal communication). It will be of interest to examine whether the expansion of specific clostridial strains in infants treated with EHCF+LGG accelerates acquisition of tolerance by fortifying epithelial barrier function.

Treatment of CMA infants with extensively hydrolyzed casein formula containing LGG resulted in the enrichment of specific strains of bacteria that are associated with butyrate production (Ferrario *et al.*, 2014). The strain-level associations were not conserved, however, between patients who became tolerant, which suggests that the extraordinary degree of interpersonal strain-level bacterial diversity observed in human populations (for example Raveh-Sadka *et al.*, 2015) may result in many different ‘tolerance-associated' microbial profiles. Our findings will inform the development of effective strategies to prevent or treat food allergy based on modulation of the intestinal microbiota.

## Figures and Tables

**Table 1:** Demographic and clinical characteristics of the study population

	*Healthy subjects*	*Patients with IgE-mediated CMA*	
		*Treated with EHCF*	*Treated with EHCF+LGG*
*N*	20	7	12
Male, *n* (%)	11 (55.0)	4 (57.1)	9 (75.0)
Age, months (±s.d.)	4.2 (1.1)	4 (0.8)	4.3 (1.3)
Weight, g (±s.d.)	6937.5 (793.2)	5607.1 (480.8)	6366.7 (1074.1)
Vaginal delivery, *n* (%)	15 (75)	4 (57.1)	7 (58.3)
Duration of exclusive breastfeeding, days (±s.d.)	14.4 (4.5)	14.8 (5.4)	15.2 (3.1)
Age of solid food introduction, months (±s.d.)	4 (0.2)	4.1 (0.4)	4.1 (0.3)

*Symptoms at the CMA onset*			
Vomiting, *n* (%)	—	4 (57.1)	8 (66.7)
Urticaria/angioedema, *n* (%)	—	3 (42.9)	4 (33.3)
Cough/wheezing, *n* (%)	—	1 (14.3)	3 (25.0)

Abbreviations: CMA, cow's milk allergy; EHCF, extensively hydrolyzed casein formula; LGG, *Lactobacillus rhamnosus* GG.
