# Supplementary material for: Lactobacillus rhamnosus GG-supplemented formula expands butyrate-producing bacterial strains in food allergic infants
Source: ISME J. 2015 Sep 22;10(3):742–50. doi: 10.1038/ismej.2015.151 (PMC4817673; doi:10.1038/ismej.2015.151)
Supplement: Supplementary Figures and Tables [file ismej2015151x1.pdf]

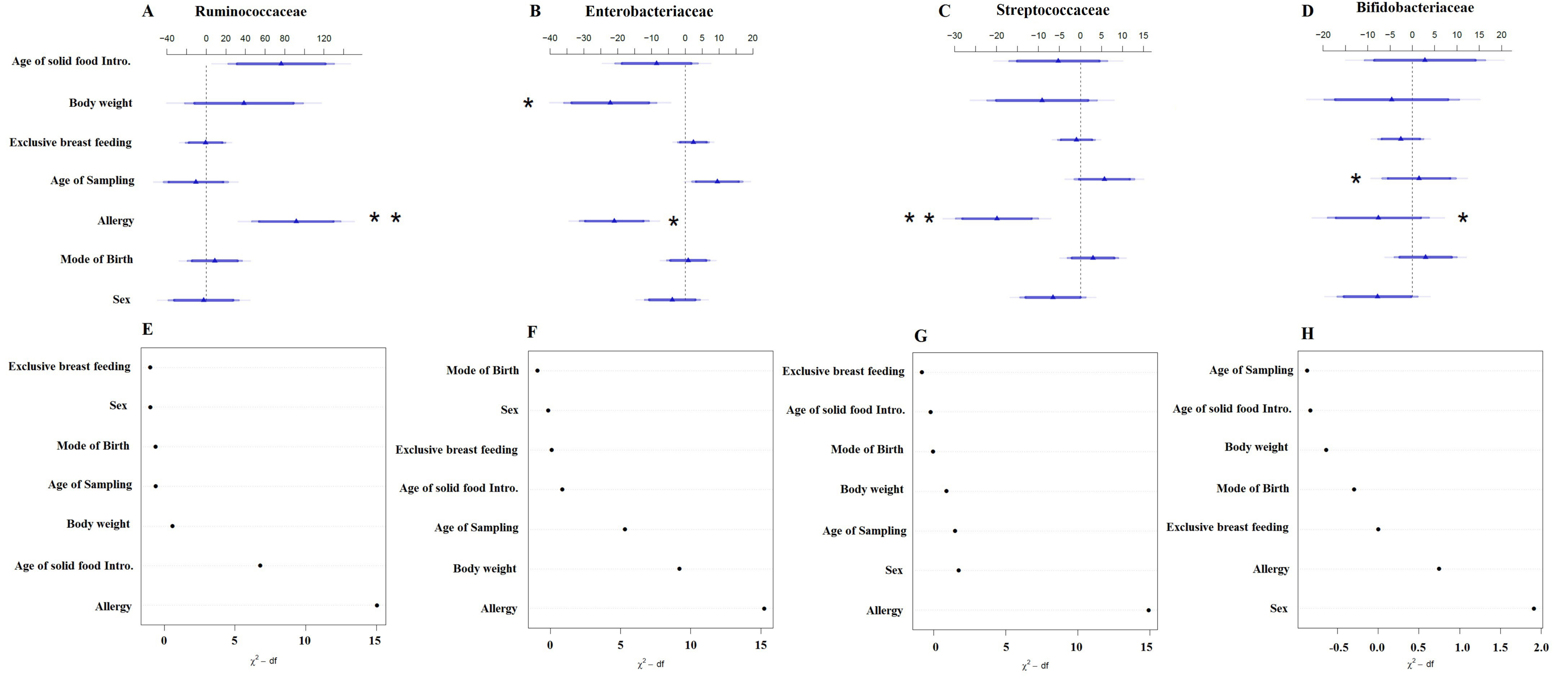
A
Ruminococcaceae
-40
0
20
40
60
80
120
Age of solid food Intro.
Body weight
Exclusive breast feeding
Age of Sampling
Allergy
Mode of Birth
Sex
* *
B
Enterobacteriaceae
-40
-30
-20
-10
0
5
10
20
*
*
C
Streptococcaceae
-30
-20
-10
-5
0
5
10
15
* *
D
Bifidobacteriaceae
-20
-10
-5
0
5
10
15
20
*
*
E
Exclusive breast feeding
Sex
Mode of Birth
Age of Sampling
Body weight
Age of solid food Intro.
Allergy
0
5
10
15
χ² – df
F
Mode of Birth
Sex
Exclusive breast feeding
Age of solid food Intro.
Age of Sampling
Body weight
Allergy
0
5
10
15
χ² – df
G
Exclusive breast feeding
Age of solid food Intro.
Mode of Birth
Body weight
Age of Sampling
Sex
Allergy
0
5
10
15
χ² – df
H
Age of Sampling
Age of solid food Intro.
Body weight
Mode of Birth
Exclusive breast feeding
Allergy
Sex
-0.5
0.0
0.5
1.0
1.5
2.0
χ² – df

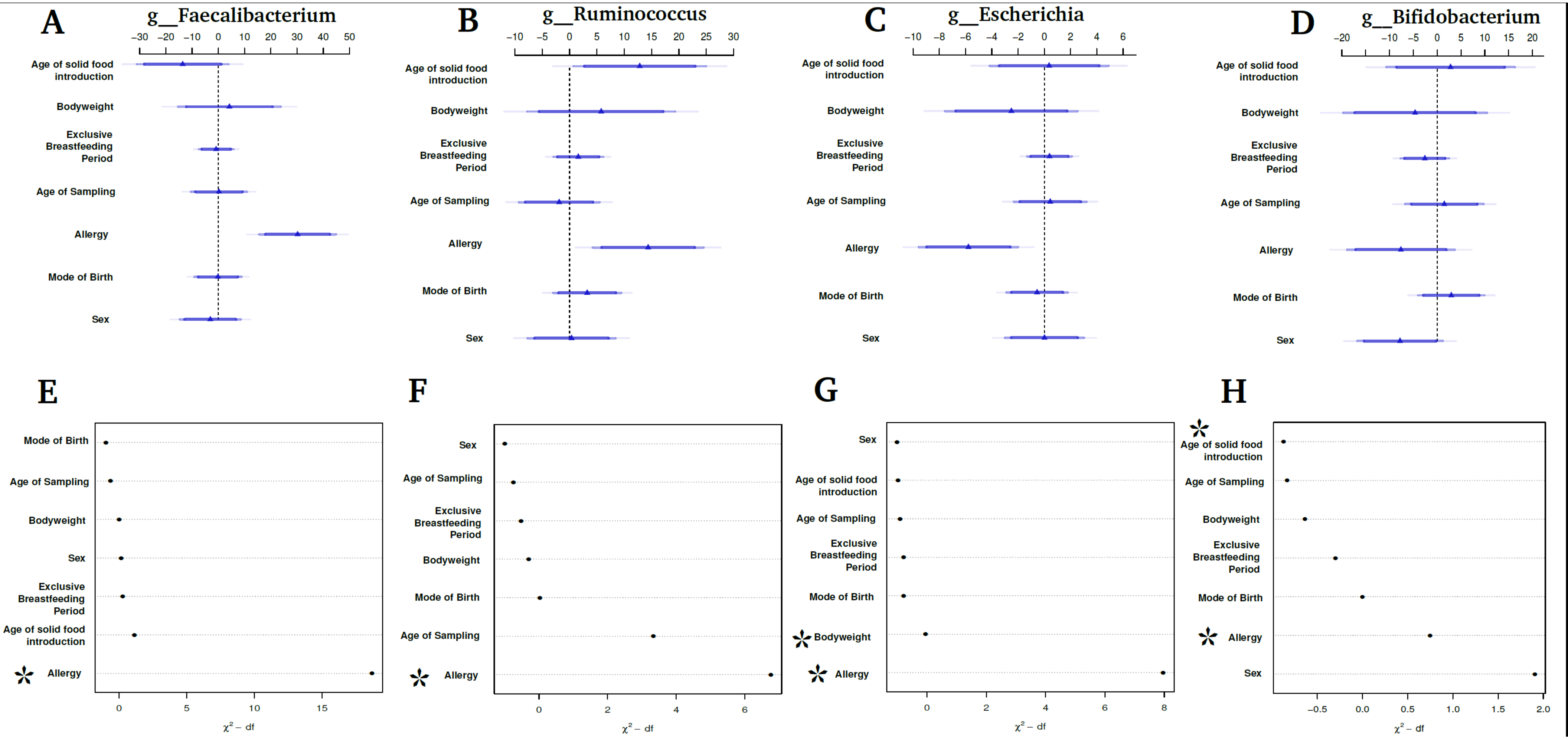
A
g__Faecalibacterium
B
g__Ruminococcus
C
g__Escherichia
D
g__Bifidobacterium
Age of solid food introduction
Bodyweight
Exclusive Breastfeeding Period
Age of Sampling
Allergy
Mode of Birth
Sex
E
F
G
H
χ² – df

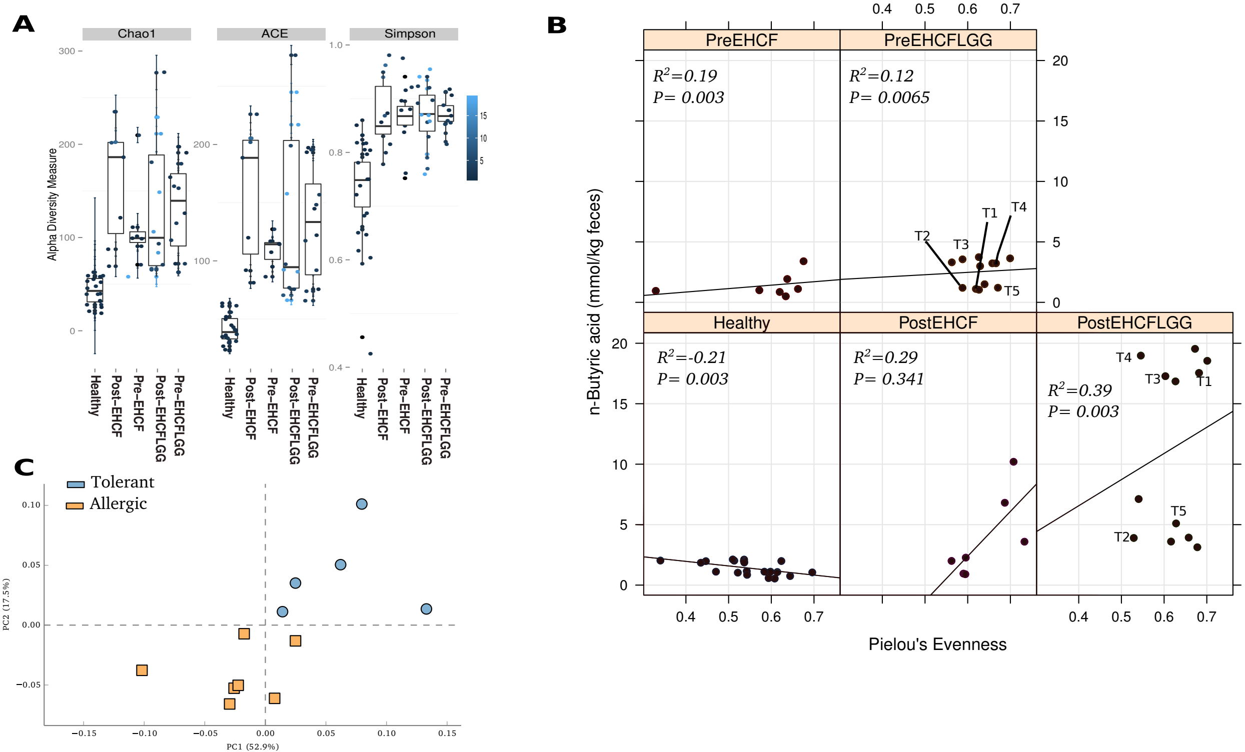
A
Chao1
ACE
Simpson
Alpha Diversity Measure
Healthy
Post-EHCF
Pre-EHCF
Post-EHCFLGG
Pre-EHCFLGG
B
PreEHCF
PreEHCFLGG
Healthy
PostEHCF
PostEHCFLGG
R^2=0.19
P= 0.003
R^2=0.12
P= 0.0065
R^2=-0.21
P= 0.003
R^2=0.29
P= 0.341
R^2=0.39
P= 0.003
T1
T2
T3
T4
T5
n-Butyric acid (mmol/kg feces)
Pielou's Evenness
C
Tolerant
Allergic
PC1 (52.9%)
PC2 (17.5%)

Sample ID	Group	Mode of Birth	Age at introduction of solid foods (months)	Age at initial sampling (months)	AgeAtSecondSampling (months)	Sex	Bodyweight (g)	Duration of exclusive breastfeeding (days)	Vomiting	UrticariaAngioedema	CoughWheezing
H_1	Healthy	Vaginal	5	6	-	F	7900	19	-	-	-
H_2	Healthy	Vaginal	4	5	-	M	8000	14	-	-	-
H_3	Healthy	Vaginal	4	5	-	M	8100	15	-	-	-
H_4	Healthy	Vaginal	4	4	-	M	6400	26	-	-	-
H_5	Healthy	Vaginal	4	5	-	F	7200	15	-	-	-
H_6	Healthy	Vaginal	4	5	-	M	7400	15	-	-	-
H_7	Healthy	Vaginal	4	5	-	M	7700	25	-	-	-
H_8	Healthy	Vaginal	4	5	-	M	7500	11	-	-	-
H_9	Healthy	Vaginal	4	5	-	F	7400	14	-	-	-
H_10	Healthy	Vaginal	4	2	-	M	5800	10	-	-	-
H_11	Healthy	Vaginal	4	2	-	M	5700	11	-	-	-
H_12	Healthy	Vaginal	4	2	-	F	5400	14	-	-	-
H_13	Healthy	Caesarean	4	3	-	M	6400	10	-	-	-
H_14	Healthy	Caesarean	4	5	-	F	7000	15	-	-	-
H_15	Healthy	Vaginal	4	4	-	F	6300	10	-	-	-
H_16	Healthy	Vaginal	4	4	-	F	6400	14	-	-	-
H_17	Healthy	Caesarean	4	4	-	F	6500	10	-	-	-
H_18	Healthy	Vaginal	4	5	-	F	6800	12	-	-	-
H_19	Healthy	Caesarean	4	4	-	M	7400	11	-	-	-
H_20	Healthy	Caesarean	4	4	-	M	7450	17	-	-	-
Pre_E_LGG_1	EHCFLGG	Vaginal	5	6	12	F	7450	17	0	1	1
Pre_E_LGG_2	EHCFLGG	Vaginal	4	5	11	M	6500	11	1	0	0
Pre_E_LGG_3	EHCFLGG	Vaginal	4	5	11	M	6800	14	1	0	0
Pre_E_LGG_4	EHCFLGG	Vaginal	4	4	10	M	6400	10	1	0	0
Pre_E_LGG_5	EHCFLGG	Vaginal	4	5	11	F	6900	15	1	0	0
Pre_E_LGG_6	EHCFLGG	Vaginal	4	5	11	M	7100	14	1	0	0
Pre_E_LGG_7	EHCFLGG	Caesarean	4	5	11	M	7000	18	1	0	0
Pre_E_LGG_8	EHCFLGG	Caesarean	4	5	11	M	7150	14	0	1	1
Pre_E_LGG_9	EHCFLGG	Caesarean	4	5	11	F	6800	15	1	0	0
Pre_E_LGG_10	EHCFLGG	Caesarean	4	2	8	M	4200	19	1	0	1
Pre_E_LGG_11	EHCFLGG	Caesarean	4	2	8	M	4300	21	0	1	0
Pre_E_LGG_12	EHCFLGG	Vaginal	4	3	9	M	5800	14	0	1	0
Pre_E_6	EHCF	Vaginal	4	3	9	M	5150	10	0	1	0
Pre_E_7	EHCF	Caesarean	4	4	10	F	5200	18	0	1	0
Pre_E_3	EHCF	Vaginal	5	5	11	M	6400	22	0	0	1
Pre_E_1	EHCF	Vaginal	4	4	10	M	5500	20	1	1	0
Pre_E_2	EHCF	Vaginal	4	3	9	M	5350	15	1	0	0
Pre_E_4	EHCF	Caesarean	4	5	11	F	6150	11	1	0	0
Pre_E_5	EHCF	Caesarean	4	4	10	F	5500	8	1	0	0

Supplementary Table 2 – Comparative relative abundance (% of total reads) and standard deviations for genera associated with different groups. For oligotype analyses, the relative abundance is percentage of oligotype reads for that group.

**Taxa**	**Healthy**	**CMA**
Ruminococcus	1.3±2.28	4.52±2.1
Faecalibacterium	0.03±0.02	7.12±8.4
Bifidobacterium	30.02±23.20	12.52±18.1
Escherichia	18.1±15.28	3.52±6.1
	**Pre EHCF with LGG**	**Post EHCF with LGG**
Blautia	1.3 ± 2.28	4.52 ± 2.1
Roseburia	2.15 ± 1.79	5.4 ± 2.07
Coprococcus	0.75 ± 0.6	1.08 ± 0.09
	**Pre EHCF**	**Post EHCF**
Rosebuira	2.09 ± 1.6	4.2 ± 2.1
	**Post EHCF**	**Post EHCF with LGG**
Rosebuira	0.45 ±0.19	1.4 ±1.9
Anaerofustis	0.00 ±0.00	0.3 ±0.02
	**Post EHCF+LGG High Butyrate**	**Post EHCF+LGG Low Butyrate**
Bacteroides	5.2 ± 0.23	13.7 ± 0.89
Faecalibacterium	8.78 ± 1.7	4.7 ± 0.3
Blautia	7.4% ± 0.3	1.3 ± 0.27
Ruminicoccus	4.5% ± 0.12	3.9 ± 0.07
Roseburia	5.02 ± 1.2	3.09 ± 0.23
	**Tolerant with <6 mmol/kg fecal butyrate (T2 & 5)**	**Tolerant with >10 mmol/kg fecal butyrate (T1, 3 & 4)**
Roseburia	1.7 ± 0.06	7.5 ± 0.36
Blautia	5.4 ± 1.02	12.35 ± 0.36
Bacteroides	18.34 ± 1.6	7.0 ± 0.96
Roseburia oligotype 2	35 ± 0.25	50.7 ± 0.3
Coprococcus oligptype 1	87 ± 0.29	92.4 ± 0.3
	**Allergic post EHCF with LGG**	**Tolerant post EHCF with LGG**
Oscillospira	4.22 ± 0.21	1.77 ± 0.94
Roseburia oligotype 2	42 ± 0.43	49 ± 1.2
Coprococcus oligptype 1	85.89 ± 6.64	90.89 ± 4.3
	**Tolerant pre EHCF with LGG**	**Tolerant post EHCF with LGG**
Roseburia oligotype 2	20.72 ± 1.5	45.72 ± 1.9
Coprococcus oligptype 1	75.304±3.5	89.70±4.6
