# Supplementary material for: Lactobacillus rhamnosus GG-supplemented formula expands butyrate-producing bacterial strains in food allergic infants
Source: ISME J. 2015 Sep 22;10(3):742–50. doi: 10.1038/ismej.2015.151 (PMC4817673; doi:10.1038/ismej.2015.151)
Supplement: Supplementary Information [file ismej2015151x2.docx]

Supplementary Figure 1. *Generalized linear model fitting analysis across significantly important taxa (Families as predicted by MetagenomeSeq) across CMA and treatment groups (EHCF and EHCF+LGG).* Upper and lower panel represent the GLM model and its ANOVA-based implementation as predicted by ‘rms’ and ‘ResourceSelection’ packages, respectively. ** *P* < 0.00001, * *P* < 0.001.

Supplementary Figure 2. *Generalized linear model fitting analysis across significantly important taxa (genera as predicted by MetagenomeSeq) across CMA and treatment groups (EHCF and EHCF+ LGG).* Upper and lower panel represent the GLM model and its ANOVA-based implementation as predicted by ‘rms’ and ‘ResourceSelection’ packages, respectively. ** *P* < 0.00001, * *P* < 0.001.

Supplementary Figure 3. *Phylogenetic diversity/evenness and butyrate production across healthy, CMA and treatment groups.* (A) box and whisker plot representing alpha diversity i.e. Shannon diversity, across all groups. Color bar intensity represents the butyrate concentration i.e. mmol/kg. (B) Linear regression analysis between phylogenetic evenness and fecal butyrate concentration across healthy CMA and treatment groups. (C) 16S rRNA based beta diversity (phylogeny independent i.e. Bray Curtis) grouping of samples from tolerant and allergic infants Post-EHCF+LGG treatment.
